# Reliability and Minimal Detectable Change Values for Predictions of Knee Forces during Gait and Stair Ascent Derived from the FreeBody Musculoskeletal Model of the Lower Limb

**DOI:** 10.3389/fbioe.2017.00074

**Published:** 2017-12-08

**Authors:** Phil D. B. Price, Conor Gissane, Daniel J. Cleather

**Affiliations:** ^1^School of Sport, Health and Applied Sciences, St. Mary’s University, Twickenham, United Kingdom

**Keywords:** tibiofemoral joint, patellofemoral joint, knee osteoarthritis, musculoskeletal modeling, validation, peak knee adduction moment, KAM, medial tibiofemoral joint load share

## Abstract

FreeBody is a musculoskeletal model of the lower limb used to calculate predictions of muscle and joint contact forces. The validation of FreeBody has been described in a number of publications; however, its reliability has yet to be established. The purpose of this study was, therefore, to establish the test–retest reliability of FreeBody in a population of healthy adults in order to add support to previous and future research using FreeBody that demonstrates differences between cohorts after an intervention. We hypothesized that test–retest estimations of knee contact forces from FreeBody would demonstrate a high intra-class correlation. Kinematic and kinetic data from nine older participants (4 men: mean age = 63 ± 11 years; 5 women: mean age = 49 ± 4 years) performing level walking and stair ascent was collected on consecutive days and then analyzed using FreeBody. There was a good level of intra-session agreement between the waveforms for the individual trials of each activity during testing session 1 (*R* = 0.79–0.97). Similarly, overall there was a good inter-session agreement within subjects (*R* = 0.69–0.97) although some subjects showed better agreement than others. There was a high level of agreement between the group mean waveforms of the two sessions for all variables (*R* = 0.882–0.997). The intra-class correlation coefficients (ICC) were very high for peak tibiofemoral joint contact forces (TFJ) and hamstring forces during gait, for peak patellofemoral joint contact forces and quadriceps forces during stair ascent and for peak lateral TFJ and the proportion of TFJ accounted for by the medial compartment during both tasks (ICC = 0.86–0.96). Minimal detectable change (MDC) of the peak knee forces during gait ranged between 0.43 and 1.53 × body weight (18–170% of the mean peak values). The smallest MDCs were found for medial TFJ share (4.1 and 5.8% for walking and stair ascent, respectively, or 4.8 and 6.7% of the mean peak values). In conclusion, the results of this study support the use of FreeBody to investigate the effect of interventions on muscle and joint contact forces at the cohort level, but care should be taken if using FreeBody at the subject level.

## Introduction

Model verification and validation are important issues within the engineering community (Oberkampf et al., 2004; Oberkampf and Barone, 2006). Verification is the process of determining that a model implementation accurately represents both the conceptual description of the model and the solution to the model (i.e., verification deals with the accuracy of the mathematical solution of the code), whereas validation is the process of determining the degree to which a model is an accurate representation of the natural system from the perspective of the intended uses of the model. When a model is “validated,” the computational results from the model are in agreement with results which are measured experimentally. Lund et al. (2012) have provided details on the verification and validation of multibody musculoskeletal models specifically. This process has since been refined in a paper by Hicks et al. (2015), which provides a “yes or no” framework for verifying and validating a model through its development. This includes developing an understanding of how the mathematical framework answers a specific question, the identification of any assumptions to reduce model complexity and how they might influence the model’s outputs, and using experimental data to identify variability, error, and uncertainty. One aspect of the evaluation of musculoskeletal models that is largely absent from the literature is a discussion of the test–retest reliability of such models (where reliability is the ability of a test to yield similar results when performed under the same conditions and a model that has high test–retest reliability is one that will yield similar results when used to analyze a subject performing a particular movement under the same conditions on two different occasions). Given that any textbook on research methods will describe that a necessary condition for a test to be valid is that it is reliable, this omission is particularly surprising.

Bioengineers are keen to use musculoskeletal models to ask “what-if” clinical research questions. In the first instance, the goal is to create musculoskeletal models that can be used to describe movement accurately at the cohort level (that is, to provide a valid and reliable description of the movement characteristics of a particular population; Cleather and Bull, 2012). For instance, researchers interested in the knee might investigate abnormal loading in those with existing knee pathology in comparison to healthy knees, and the assessment of knee forces before and after an intervention. Ultimately, the aspiration of this field is to create models which can be used at a subject specific level in order to evaluate individual patients and to inform the planning of therapeutic, medical and surgical treatments (Cleather and Bull, 2012). For instance, these data could be used to effectively influence clinical decision-making during knee assessment, providing practitioners with sufficient information to create appropriate rehabilitation strategies, leading to more effective treatments for common knee pathologies, reducing pathological symptoms and improving quality of life. However, before musculoskeletal models can be used for this purpose, their reliability needs to be investigated. The determination of the reliability of a musculoskeletal model is essential to assess if any detectable changes in model outputs are from genuine adaptations caused by an intervention, or are caused by the level of error associated with the model, the experimental procedures during data collection, or day to day variation. Failure to determine the level of error associated with a musculoskeletal model may lead to misinterpretation of the data, thus leading to the development of inappropriate clinical decisions and rehabilitation strategies.

The results of musculoskeletal model simulations will be sensitive to measurement error [e.g., instrumental error (Chiari et al., 2005), soft tissue artifact (Leardini et al., 2005), or misplacement of markers (Croce et al., 2005)], despite the best efforts of the researcher to keep this minimal. To ensure these outputs can be used to provide an understanding of the musculoskeletal system and have the potential to be used to influence clinical decisions, the level of error must be determined. The Minimal Detectable Change (MDC) is a distribution-change index which represents a difference or “real change” and is not attributed to measurement error or chance and that has been employed to assess change in patient populations (Fulk and Echternach, 2008; Steffen and Seney, 2008; Wagner et al., 2008). However, to date, only Gardinier et al. (2013) and Barrios and Willson (2016) have published the MDCs for the tibiofemoral joint contact force (TFJ) predicted by their musculoskeletal models during gait and MDCs for muscle forces have not been reported.

FreeBody is a publicly available musculoskeletal model of the lower limb (Cleather and Bull, 2015), the development and validation of which has been described extensively within the literature (Cleather and Bull, 2010a,b, 2011a,b; Cleather et al., 2011a,b; Southgate et al., 2012; Ding et al., 2016; Price et al., 2016). In particular, the sensitivity of the model to its assumptions (for instance, the inverse dynamics methodology, the musculoskeletal geometry data set employed, and the degrees of freedom of the joints; Cleather and Bull, 2010a,b, 2011b) and to measurement error (e.g., error in the measurement of shank segment axial rotation; Southgate et al., 2012) have been determined and the predictions of the model during activities of daily living and more dynamic activities have been compared to experimental measurements including electromyography and joint contact forces measured by telemetry from instrumented prostheses (Cleather and Bull, 2015; Ding et al., 2016; Price et al., 2016). However, the inter-session reliability of FreeBody is currently unknown. In recent years, FreeBody has been used to evaluate the effect of acute and chronic exercise interventions and the model has demonstrated differences in muscle and joint contact forces pre and post intervention(Czasche et al., 2017; Parr et al., 2017). The aim of this study was, therefore, to assess the reliability of the FreeBody model in order to add support to this previous research and to evaluate the use of FreeBody at the cohort level. We hypothesized that test–retest estimations of knee contact forces from FreeBody based on data collected on consecutive days would demonstrate a high intra-class correlation. A secondary goal was to evaluate the use of FreeBody at the subject level and to determine its MDCs. This information will be useful in understanding the path toward a clinical tool that can be used at the subject-specific level.

## Materials and Methods

For this test–retest reliability study, participants were required to attend St Mary’s University laboratory for two data collection sessions separated by 24 h. During each testing session, kinematic and kinetic data were collected during gait and stair ascent. These data were then processed using FreeBody in order to generate predictions of muscle and knee joint contact forces, which were then analyzed to determine the reliability of the model.

### Participants

Participants consisted of four males and five females (Table 1). Prior to testing, all participants were familiarized with the demands of the study and then completed an informed consent form and a health questionnaire. All participants were free from injury of the right lower limb during the last 6 months and did not feel pain in their right knee (VAS score = 0) while performing the test protocol. This population was chosen based upon the assumption that their gait should not vary substantially between test sessions, and thus the reliability statistics calculated should predominantly represent the test—retest reliability of the model and data collection procedure only. All participants refrained from strenuous exercise and caffeine ingestion 24 hours prior to testing to minimize the effect of existing training fatigue and stimulatory effects on the data collected. All procedures were approved by the St Mary”s University ethics committee.

**Table 1:** Participant characteristics.

Participant	Gender	Age (years)	Height (cm)	Body mass (kg)	
				T1	T2
RS1	M	72	175	68.6	68.6
RS2	M	59	181	90.6	90.8
RS3	M	70	175	71.0	70.6
RS4	M	49	185	60.3	60.3
Mean		63	179	72.6	72.6
SD		11	5	12.8	12.9
RS5	F	46	165	51.0	50.9
RS6	F	45	154	44.6	44.8
RS7	F	55	171	86.8	87.7
RS8	F	50	160	45.2	45.9
RS9	F	50	152	51.9	51.5
Mean		49	160	55.9	56.1
SD		4	8	17.6	17.9

*M, male; F, female; T1, test session one; T2, test session two*.

### Instrumentation

Motion capture data were collected using an 11 camera Vicon motion capture system (Vicon MX System, Vicon Motion Systems Ltd., Oxford, UK) at a sampling rate of 200 Hz. Ground reaction force data were collected using a recessed 600 mm × 900 mm Kistler 9287BA force plate (Kistler Instruments Ltd., Hook, UK) that was synchronized with Vicon at a sampling rate of 1,000 Hz.

### Procedures

Eighteen 25-mm reflective markers were placed on landmarks of the right lower limb and pelvis in accordance with the FreeBody guidelines (Cleather and Bull, 2015). The reflective markers were attached to the pre-determined landmarks by the principal researcher every session. All participants wore shorts which would not obstruct the reflective markers from motion capture. Initially, an anatomical calibration trial was obtained with the participant standing motionless in the anatomical position. The participants were then required to perform two exercises which were representative of activities of daily living; level walking; and stair ascent. Each exercise was practiced by the participant until they felt comfortable performing the activity. Three successful trials were collected for each activity.

First, participants performed level walking. The aim was to ensure that data collection occurred on the fourth step of gait (we found that four steps was adequate to achieve a natural gait pattern). Starting position was determined by standing on the force plate and taking four steps back and marking the start position. Participants repeated walking trials until they felt they performed a comfortable walking gait without altering their stance to make contact with the force plate. Participants were instructed to walk at a self-selected pace and look forward so they would not focus on making contact with the force plate. A trial was deemed successful when the participant made contact with force plate with no obvious attempt to alter their stride during gait.

**Figure 1:**
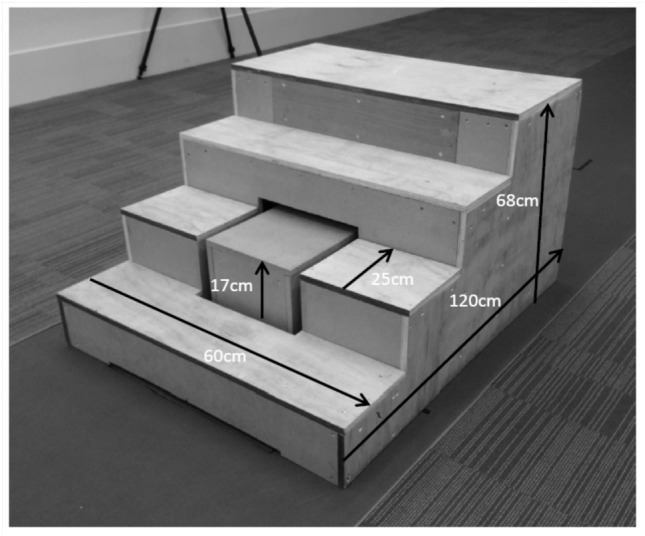
The staircase employed for stair ascent in this study.

Second, participants were required to perform stair ascent at a self-selected pace. A custom-built staircase (Figure 1) was created to the specification of Aminaka et al. (2011). The second step of the staircase was replaced by a separate box, which was placed directly on the force platform. An opening was cut into the staircase to receive the box, with a 3-cm border of space in order to avoid any contact between the staircase and the box, thus minimizing noise artifact when the participant was in contact with the other stairs. Participants were instructed not to focus on the second step to avoid any deliberate maneuver to make contact with it. Each participant was encouraged to perform stair ascent in the way they would do in everyday life. A trial was deemed successful when the participant made clear contact with the box representing the second step and no accidental contact was made between the stairs and any of the reflective markers. The center of pressure of the foot on the step was calculated based on the assumption that the sum of the forces and moments acting on the box was 0. First, the force exerted by the foot on the step was calculated based upon the ground reaction force and the weight of the box. The center of pressure of the foot on the step was calculated such that the moment acting on the box around its center of mass was 0, and that the ground reaction force acted through the center of pressure measured by the force plate.

### Musculoskeletal Model (FreeBody)

All raw kinetic, kinematic, and EMG data were synchronized at 200 Hz and pre-processed into the input format of FreeBody. Kinetic data and kinematic data were filtered using a fourth order low-pass Butterworth filter with a cutoff frequency of 15 and 6 Hz, respectively prior to being processed using FreeBody.

The FreeBody model consists of five rigid segments of the foot, shank, thigh, pelvis, and patella. The location and orientation of the segments are determined in the fixed global coordinate frame from the reflective markers. No kinematic constraints are applied to the foot, shank, thigh, or pelvis, and so each of these segments have six degrees of freedom. The position and orientation of the patella is calculated based upon the position of the thigh and the knee flexion angle (0 degrees of freedom).

Once the location and orientation of each segment for every frame has been established, the musculoskeletal geometry can be added. The origin, insertion, and path of 163 muscles, the patellar tendon, and 14 ligaments are defined for each segment in the local coordinate system using the Klein Horsman cadaver data set (Horsman et al., 2007). The mass, center of mass, and inertial properties of each segment are determined using the data provided by De Leva (1996).

The marker trajectories of the segments and the ground reaction forces obtained during data collection provide the kinematic and kinetic data, respectively. These data, along with the musculoskeletal geometry of the model provide the parameters for the development of the indeterminate equations of motions, which govern the movement of the lower limb (Eq. 1). The most physiologically likely solution to the equations of motion is found by minimizing the sum of the muscle stresses and ligament stresses cubed (Eq. 2) and is derived from the previous work of Crowninshield and Brand (1981) and Raikova (2009).

(1) $$\begin{array}{ll} & \left(\begin{array}{cccc} \begin{array}{ccc} {\hat{p}}_{1}^{1} & \ldots & {\hat{p}}_{M}^{1}\\ {\hat{p}}_{1}^{2} & \ldots & {\hat{p}}_{M}^{2}\\ {\hat{p}}_{1}^{3} & \ldots & {\hat{p}}_{M}^{3} \end{array}\; & \begin{array}{c} {\hat{p}}_{\mathrm{pt}}^{1}\\ {\hat{p}}_{\mathrm{pt}}^{2}\\ {\hat{p}}_{\mathrm{pt}}^{3} \end{array}\; & \begin{array}{ccc} {\hat{q}}_{1}^{1} & \ldots & {\hat{q}}_{N}^{1}\\ {\hat{q}}_{1}^{2} & \ldots & {\hat{q}}_{N}^{2}\\ {\hat{q}}_{1}^{3} & \ldots & {\hat{q}}_{N}^{3} \end{array}\; & \begin{array}{ccccc} -I_{3\times 3} & E_{3\times 3} & E_{3\times 3} & E_{3\times 3} & E_{3\times 3}\\ I_{3\times 3} & -I_{3\times 3} & -I_{3\times 3} & E_{3\times 3} & E_{3\times 3}\\ E_{3\times 3} & I_{3\times 3} & I_{3\times 3} & -I_{3\times 3} & I_{3\times 3} \end{array}\\ \begin{array}{ccc} {\hat{r}}_{1}^{1}\times {\hat{p}}_{1}^{1} & \ldots & {\hat{r}}_{M}^{1}\times {\hat{p}}_{M}^{1}\\ {\hat{r}}_{1}^{2}\times {\hat{p}}_{1}^{2} & \ldots & {\hat{r}}_{M}^{2}\times {\hat{p}}_{M}^{2}\\ {\hat{r}}_{1}^{3}\times {\hat{p}}_{1}^{3} & \ldots & {\hat{r}}_{M}^{3}\times {\hat{p}}_{M}^{3} \end{array}\; & \begin{array}{c} {\hat{r}}_{\mathrm{pt}}^{1}\times {\hat{p}}_{\mathrm{pt}}^{1}\\ {\hat{r}}_{\mathrm{pt}}^{2}\times {\hat{p}}_{\mathrm{pt}}^{2}\\ {\hat{r}}_{\mathrm{pt}}^{3}\times {\hat{p}}_{\mathrm{pt}}^{3} \end{array}\; & \begin{array}{ccc} {\hat{s}}_{1}^{1}\times {\hat{q}}_{1}^{1} & \ldots & {\hat{s}}_{N}^{1}\times {\hat{q}}_{N}^{1}\\ {\hat{s}}_{1}^{2}\times {\hat{q}}_{1}^{2} & \ldots & {\hat{s}}_{N}^{2}\times {\hat{q}}_{N}^{2}\\ {\hat{s}}_{1}^{3}\times {\hat{q}}_{1}^{3} & \ldots & {\hat{s}}_{N}^{3}\times {\hat{q}}_{N}^{3} \end{array}\; & \begin{array}{ccccc} E_{3\times 3} & E_{3\times 3} & E_{3\times 3} & E_{3\times 3} & E_{3\times 3}\\ {\tilde{d}}^{2} & -{\tilde{h}}_{1}^{2} & -{\tilde{h}}_{2}^{2} & E_{3\times 3} & E_{3\times 3}\\ E_{3\times 3} & {\tilde{d}}_{1}^{3} & {\tilde{d}}_{2}^{3} & E_{3\times 3} & {\tilde{f}}^{\;3} \end{array}\\ \begin{array}{ccc} {\hat{p}}_{1}^{\mathrm{pat}} & \ldots & {\hat{p}}_{M}^{\mathrm{pat}} \end{array}\; & {\hat{p}}_{\mathrm{pt}}^{\mathrm{pat}}\; & E_{3\times N}\; & \begin{array}{ccccc} E_{3\times 3} & E_{3\times 3} & E_{3\times 3} & E_{3\times 3} & -I_{3\times 3} \end{array}\\ \begin{array}{ccc} \rho_{1} & \ldots & \rho_{M} \end{array}\; & -1\; & E_{1\times N}\; & \begin{array}{ccccc} E_{1\times 3} & E_{1\times 3} & E_{1\times 3} & E_{1\times 3} & E_{1\times 3} \end{array} \end{array}\right)\;\left(\begin{array}{c} \begin{array}{c} F_{1}\\ \vdots \\ F_{M} \end{array}\\ F_{\mathrm{pt}}\\ \begin{array}{c} L_{1}\\ \vdots \\ L_{N} \end{array}\\ \begin{array}{c} {\hat{R}}^{1}\\ {\hat{R}}_{1}^{2}\\ {\hat{R}}_{2}^{2}\\ {\hat{R}}^{3} \end{array}\\ {\hat{R}}^{\mathrm{pat}} \end{array}\right)\\ & \;=\left(\begin{array}{c} \begin{array}{c} m^{1}\left({\hat{a}}^{1}-\hat{g}\right)-{\hat{S}}^{0}\\ m^{2}\left({\hat{a}}^{2}-\hat{g}\right)\\ m^{3}\left({\hat{a}}^{3}-\hat{g}\right) \end{array}\\ \begin{array}{c} m^{1}{\hat{c}}^{1}\times \left({\hat{a}}^{1}-\hat{g}\right)+Y_{3\times 3}^{1}{\ddot{\hat{\varphi }}}^{1}+{\dot{\hat{\varphi }}}^{1}\times Y_{3\times 3}^{1}{\dot{\hat{\varphi }}}^{1}-{\hat{d}}^{1}\times {\hat{S}}^{0}-M^{0}\\ m^{2}{\hat{c}}^{2}\times \left({\hat{a}}^{2}-\hat{g}\right)+Y_{3\times 3}^{2}{\ddot{\hat{\varphi }}}^{2}+{\dot{\hat{\varphi }}}^{2}\times Y_{3\times 3}^{2}{\dot{\hat{\varphi }}}^{2}\\ m^{3}{\hat{c}}^{3}\times \left({\hat{a}}^{3}-\hat{g}\right)+Y_{3\times 3}^{3}{\ddot{\hat{\varphi }}}^{3}+{\dot{\hat{\varphi }}}^{3}\times Y_{3\times 3}^{3}{\dot{\hat{\varphi }}}^{3} \end{array}\\ E_{3\times 1}\\ 0 \end{array}\right) \end{array}$$

(2) $${\text{min}}_{F_{i}},\;L_{j}\;J=\sum_{i=1}^{M}\;{\left(\frac{F_{i}}{F\text{ma}{\text{x}}_{i}}\right)}^{3}+\sum_{j=1}^{N}\;{\left(\frac{L_{i}}{L\text{ma}{\text{x}}_{i}}\right)}^{3}$$

where:

**Table:** 

${\hat{a}}^{k}$	linear acceleration of the center of mass of segment *k*
${\hat{c}}^{k}$	vector from center of rotation of joint at proximal end of segment *k* to center of mass of segment *k*
${\hat{d}}^{k}$	vector from center of rotation of joint at proximal end of segment *k* to center of rotation joint at distal end of segment *k*
${\tilde{d}}^{k}$	skew-symmetric matrix of vector ${\tilde{d}}^{k}$
${\tilde{d}}_{l}^{3}$	skew-symmetric matrix of vector from center of rotation of hip to tibiofemoral joint contact *l*
*E*_3×3_	3 × 3 matrix of 0s
${\tilde{f}}^{\;3}$	skew-symmetric matrix of vector from center of rotation of hip to contact point of patella with the femur
*F_i_*	magnitude of force in muscle *i*
$F\text{ma}{\text{x}}_{i}$	maximum possible force in muscle *i* (upper bound)
$\hat{g}$	acceleration due to gravity
${\tilde{h}}_{l}^{2}$	skew-symmetric matrix of vector from center of rotation of knee to tibiofemoral joint contact *l*
*i*	muscle number
*I*_3×3_	3 × 3 identity matrix
*j*	ligament number
*J*	cost function
*k*	segment number
*L_j_*	magnitude of force in ligament *j*
$L\text{ma}{\text{x}}_{j}$	maximum possible force in ligament *j* (upper bound)
*m^k^*	mass of segment *k*
*M*	total number of muscles
*N*	total number of ligaments
${\hat{p}}_{i}^{k}$	unit vector representing the line of action of force created by muscle *i* that acts on segment *k* (0 if muscle does not insert on segment *k*)
*pat*	Patella
*pt*	patellar tendon
${\hat{q}}_{j}^{k}$	unit vector representing the line of action of force created by ligament *j* that acts on segment *k* (0 if ligament does not insert on segment *k*)
${\hat{r}}_{i}^{k}$	vector from center of rotation of joint at proximal end of segment *k* to point of action of muscle *i* on segment *k* (0 if muscle does not insert on segment *k*)
${\hat{R}}^{k}$	vector representing *x, y*, and *z* components of reaction force acting at proximal end of segment *k*
${\hat{R}}_{l}^{k}$	vector representing *x, y*, and *z* components of reaction force *l* acting at proximal end of segment *k*
${\hat{s}}_{j}^{k}$	vector from center of rotation of joint at proximal end of segment *k* to point of action of ligament *j* on segment *k* (0 if ligament does not insert on segment *k*)
$-{\hat{S}}^{k}$	inter-segmental force acting on proximal end of segment *k*
$-{Ŵ}^{k}$	inter-segmental moment acting on proximal end of segment *k*
$Y_{3\times 3}^{k}$	inertia tensor of segment *k*
ρ*_i_*	ratio of patella to quadriceps tendon forces for muscle *i* (0 if the muscle is not part of the quadriceps muscle group)
${\dot{\hat{\varphi }}}^{k}$	angular velocity of segment *k*
${\ddot{\hat{\varphi }}}^{k}$	angular acceleration of segment *k*

### Data Analysis

Data included for analysis were when the participant was in contact with the ground (stance phase). These data were then spline fitted to 100 data points to represent a percentage of stance. The following waveform comparisons were then made:

1. Within-subject comparison of the 3 trials of both walking and stair ascent for data collection session (T1) only. For each subject, waveforms for the variables of interest were compared for trial 1 versus trial 2, trial 1 versus trial 3, and trial 2 versus trial 3 by calculating correlation coefficients (*R*) and root mean square errors (RMS). A mean *R* and RMS was calculated for each subject and each activity, and then the across subject mean for each activity was calculated.
2. Means for each activity, for each subject and for T1 and test session 2 (T2) were then calculated based on the three trials. For each subject and activity, the waveforms for T1 and T2 were compared.
3. Group means and confidence intervals were calculated for data collection sessions T1 and T2 for each task and compared.

The intra-class correlation coefficient (ICC), standard error of the mean (SEM), and MDC were calculated for the peaks of the following variables: total, medial, and lateral TFJ, proportion of TFJ accounted for by medial TFJ (%), patellofemoral joint contact force (PFJ), and quadriceps and hamstring muscle forces. In this study, the peak total TFJ was taken simply to be the arithmetic sum of the magnitudes of the medial and lateral TFJ in order that the sum of the medial and lateral TFJ shares summed to 100%. The quadriceps muscle forces were the summation of muscle force from the vastus medialis, vastus intermedialis, and vastus lateralis. Similarly, hamstring muscle forces were the summation of the bicep femoris long head, semitendinosus, and semimembranosus. ICC values were calculated using SPSS (Version 22, Chicago, IL, USA). SEM was calculated using Eq. 3:

(3) $$\text{SEM}=\text{SD}\times \sqrt{\left(1-\text{ICC}\right)}$$

where SD is the standard deviation of the data collected during T1. Using the SEM value from Eq. 1, MDCs were subsequently calculated using Eq. 4:

(4) $$\text{MDC}=\text{SEM}\times 1.96\times \sqrt{2}$$

where 1.96 represents 95% level of confidence, and multiplying by $\sqrt{2}$ provides additional uncertainty to compensate for different scores of measurements from two different time points.

## Results

**Figure 2:**
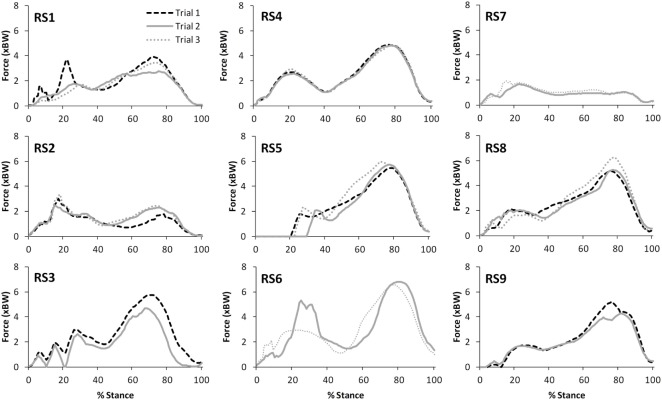
A comparison of the subject specific variation (between the 3 trials within test session 1) in predictions of medial tibiofemoral joint contact forces during gait. Note only 2 trials were available for subjects RS3, RS6, RS7, and RS9.

**Figure 3:**
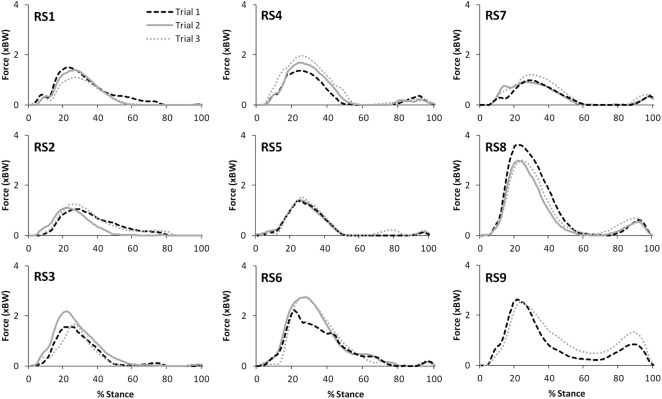
A comparison of the subject specific variation (between the three trials within test session 1) in predictions of patellofemoral joint contact forces during stair ascent. Note only two trials were available for subject RS9.

Table 2 presents the mean (across all subjects) of the mean correlations and RMS values between the waveforms for the three trials of gait and stair ascent performed in T1 (i.e., the mean of the comparisons of trial 1 to 2, trial 1 to 3, and trial 2 to 3). There was a strong correlation between all measures for both walking (mean values: *R* = 0.81–0.93) and stair ascent (mean values: *R* = 0.79–0.97). Figures 2 and 3 present a comparison of these waveforms across subjects for medial TFJ during walking (Figure 2) and PFJ during stair ascent (Figure 3). It should be noted that for a limited number of trials the optimization routine was not able to find a satisfactory solution (3 trials out of 54) and data were missing for a further 2 trials—these 5 trials were omitted from the analysis.

**Table 2:** Within-subject agreement between waveforms for the three individual trials of both gait and stair ascent performed within test session 1 [figures presented are the across subject means, of the within-subject means of the correlation coefficients (*R*) and root mean square errors (RMS) for the comparison of trial 1 to trial 2, trial 1 to trial 3, and trial 2 to trial 3].

Variable	Gait		Stair ascent	
	*R*	RMS	*R*	RMS
Peak total TFJ (×BW)	0.90	0.60	0.86	0.57
Peak lateral TFJ (×BW)	0.82	0.24	0.81	0.20
Peak medial TFJ (×BW)	0.93	0.51	0.89	0.44
Peak medial TFJ share (%)	0.81	7.65	0.92	7.10
Peak PFJ (×BW)	0.86	0.12	0.96	0.23
Peak quadriceps force (×BW)	0.87	0.11	0.97	0.19
Peak hamstrings force (×BW)	0.84	0.36	0.79	0.31

*TFJ, tibiofemoral joint contact force, PFJ, patellofemoral joint contact force, BW, body weight*.

**Figure 4:**
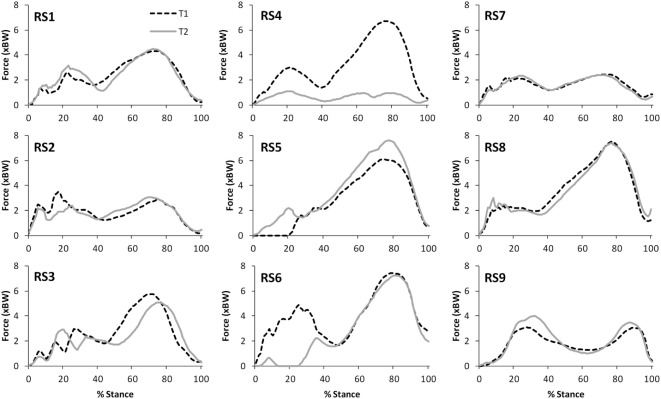
A comparison of the subject specific variation (between test sessions 1 and 2) in predictions of tibiofemoral joint contact forces during gait.

**Figure 5:**
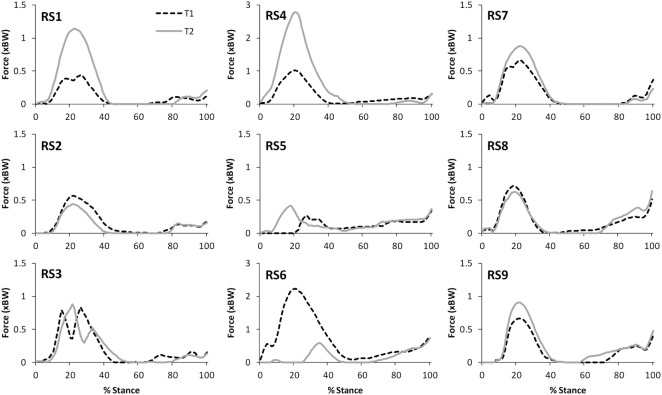
A comparison of the subject specific variation (between test sessions 1 and 2) in predictions of patellofemoral joint contact forces during gait.

**Figure 6:**
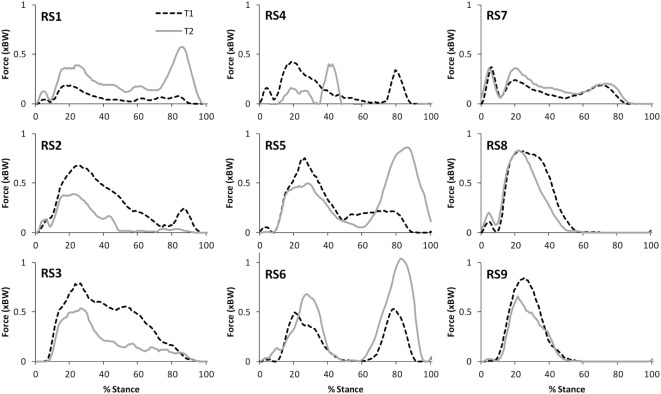
A comparison of the subject specific variation (between test sessions 1 and 2) in predictions of hamstring forces during stair ascent.

The correlations between the mean waveforms (mean of trials 1–3) within subjects for T1 and T2 are presented in Table 3 (walking) and Table 4 (stair ascent). Again, there was generally a strong correlation for all variables in both walking (mean values: *R* = 0.69–0.91) and stair ascent (mean values: *R* = 0.71–0.97). However, for some variables, there was considerable between-subject variation in the correlation between test sessions. Some examples of this are depicted in Figures 4–6.

**Table 3:** Within-subject agreement (correlation coefficients) between test sessions 1 and 2 for the mean waveforms of trials 1 to 3 for gait.

Participant	TFJ				PFJ	Quadriceps	Hamstrings
	Total	Lateral	Medial	Medial share			
RS1	0.94	0.49	0.96	0.84	0.97	0.99	0.88
RS2	0.77	0.83	0.79	0.95	0.96	0.97	0.79
RS3	0.80	0.53	0.83	0.85	0.81	0.84	0.69
RS4	0.74	0.35	0.85	0.34	0.97	0.97	0.58
RS5	0.97	0.87	0.98	0.68	0.12	0.23	0.99
RS6	0.79	0.33	0.83	0.80	0.00	0.24	0.95
RS7	0.96	0.95	0.98	0.95	0.98	0.99	0.85
RS8	0.98	0.84	0.97	0.86	0.95	1.00	0.98
RS9	0.98	0.96	0.98	0.94	0.98	1.00	0.95
Mean	0.88	0.69	0.91	0.80	0.75	0.80	0.85
SD	0.10	0.26	0.08	0.19	0.39	0.33	0.14

*TFJ, tibiofemoral joint contact force; PFJ, patellofemoral joint contact force*.

**Table 4:** Within-subject agreement (correlation coefficients) between test sessions 1 and 2 for the mean waveforms of trials 1 to 3 for stair ascent.

Participant	TFJ				PFJ	Quadriceps	Hamstrings
	Total	Lateral	Medial	Medial share			
RS1	0.91	0.92	0.84	0.87	0.99	0.99	0.54
RS2	0.89	0.92	0.88	0.91	0.99	0.99	0.85
RS3	0.93	0.81	0.94	0.93	0.98	0.97	0.87
RS4	0.70	0.77	0.87	0.89	0.94	0.92	0.34
RS5	0.84	0.48	0.71	0.63	0.99	1.00	0.16
RS6	0.96	0.93	0.94	0.97	0.96	0.96	0.78
RS7	0.89	0.85	0.96	0.90	0.93	0.94	0.91
RS8	0.98	0.95	0.99	0.96	0.99	0.99	0.94
RS9	0.95	0.86	0.96	0.95	0.98	0.98	0.98
Mean	0.89	0.83	0.90	0.89	0.97	0.97	0.71
SD	0.09	0.14	0.09	0.10	0.02	0.03	0.29

*TFJ, tibiofemoral joint contact force; PFJ, patellofemoral joint contact force*.

**Figure 7:**
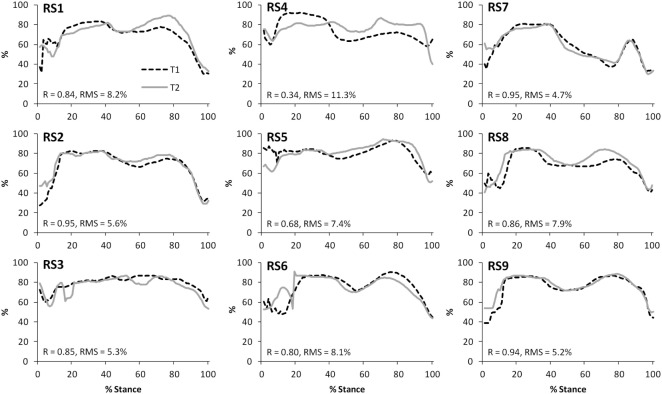
A comparison of the subject-specific variation (between test sessions 1 and 2) in predictions of the proportion of the tibiofemoral joint contact force accounted for by the medial compartment during gait. Correlation coefficients (*R*) and root mean square errors (RMS) are also presented.

The test–retest reliability data for the peak values found in this study is described in Tables 5 and 6. During gait, ICC scores were high for the TFJ (0.86–0.96) and hamstring forces (0.92), but low for the PFJ and quadriceps forces (<0.3). This resulted in higher MDC values for the PFJ and quadriceps forces (1.53–1.55 × BW) in comparison to the TFJ (0.43–0.99 × BW). Peak knee flexion occurred approximately at 24% of stance, and achieved an ICC of 0.86 and a MDC of 9.2°. In contrast to walking, for stair ascent ICC scores were lower for TFJ (0.03–0.66) and hamstring forces (0.50), but much higher for PFJ and quadriceps forces (0.92). Subsequently, MDC values were higher for TFJ (1.22–1.93 × BW) and hamstring forces (0.45 × BW), and lower for PFJ and quadriceps forces (0.50–0.51 × BW). The smallest MDC (as a percentage of the mean peak score) was for medial TFJ load share (as a percentage of total TFJ) during gait. The individual test–retest data for this variable are presented in Figure 7.

**Table 5:** Test–retest reliability measures of peak values for nine participants during level walking [mean ± SD, intra-class correlation coefficient (ICC), SEM, and minimal detectable change (MDC)].

Variable	T1	T2	ICC	SEM	MDC
Peak total TFJ (×BW)	5.43 ± 1.75	5.47 ± 1.87	0.96	0.35	0.97
Peak lateral TFJ (×BW)	1.45 ± 0.42	1.22 ± 0.40	0.86	0.15	0.43
Peak medial TFJ (×BW)	4.44 ± 1.54	4.61 ± 1.71	0.95	0.36	0.99
Peak medial TFJ share (%)	86.9 ± 4.2	87.0 ± 4.2	0.87	1.5	4.1
Peak PFJ (×BW)	0.83 ± 0.56	0.98 ± 0.71	0.22	0.55	1.53
Peak quadriceps force (×BW)	0.83 ± 0.52	1.01 ± 0.82	0.30	0.56	1.55
Peak hamstrings force (×BW)	1.20 ± 0.51	1.07 ± 0.64	0.92	0.16	0.45
Peak knee flexion (^°^)	16.7 ± 7.9	17.9 ± 10.1	0.86	3.3	9.2

*TFJ, tibiofemoral joint contact force; PFJ, patellofemoral joint contact force; BW, body weight; T1, test 1; T2, test 2*.

**Table 6:** Test–retest reliability measures of peak values for nine participants during stair ascent [mean ± SD, intra-class correlation coefficient (ICC), SEM, and minimal detectable change (MDC)].

Variable	T1	T2	ICC	SEM	MDC
Peak total TFJ (×BW)	3.80 ± 1.33	4.04 ± 0.96	0.62	0.70	1.93
Peak lateral TFJ (×BW)	1.21 ± 0.57	1.03 ± 0.29	0.03	0.44	1.22
Peak medial TFJ (×BW)	3.07 ± 1.34	3.35 ± 1.04	0.66	0.69	1.90
Peak medial TFJ share (%)	86.1 ± 6.6	86.4 ± 7.2	0.90	2.1	5.8
Peak PFJ (×BW)	1.92 ± 0.77	1.72 ± 0.57	0.92	0.19	0.51
Peak quadriceps force (×BW)	1.72 ± 0.76	1.52 ± 0.58	0.92	0.18	0.50
Peak hamstrings force (×BW)	0.60 ± 0.23	0.63 ± 0.24	0.50	0.16	0.45

*TFJ, tibiofemoral joint contact force; PFJ, patellofemoral joint contact force; BW, body weight; T1, test 1; T2, test 2*.

**Figure 8:**
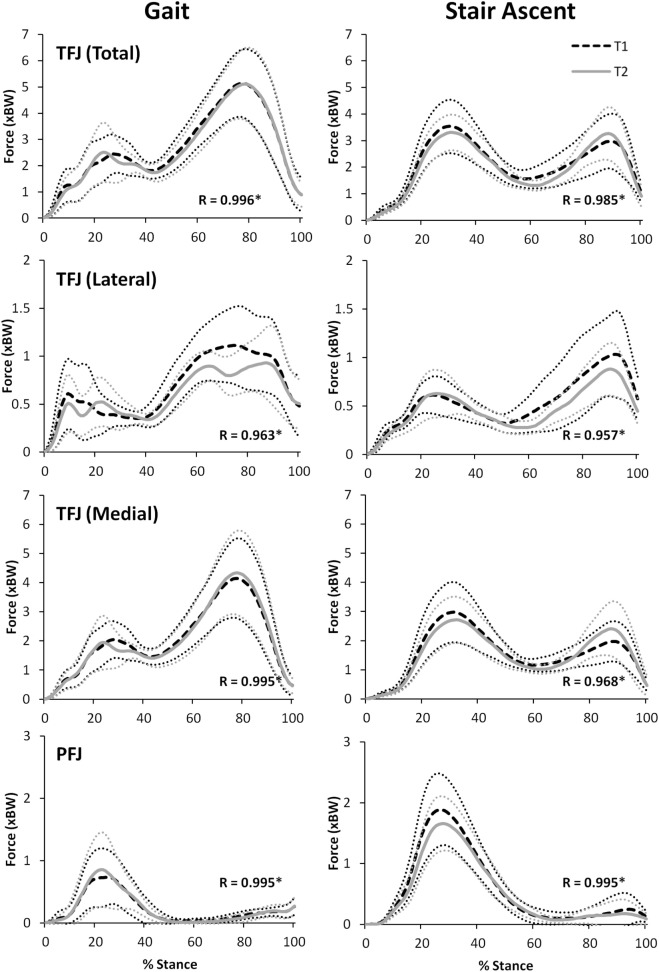
A comparison of group mean knee joint contact forces (TFJ, tibiofemoral joint contact force; PFJ, patellofemoral joint contact force) from two data collection sessions separated by 24 h. 95% confidence intervals for session one and two are represented by the thin, dotted black and grey lines, respectively. Correlation coefficients (*R*) are also included where * indicates a significant correlation (*p* < 0.05).

**Figure 9:**
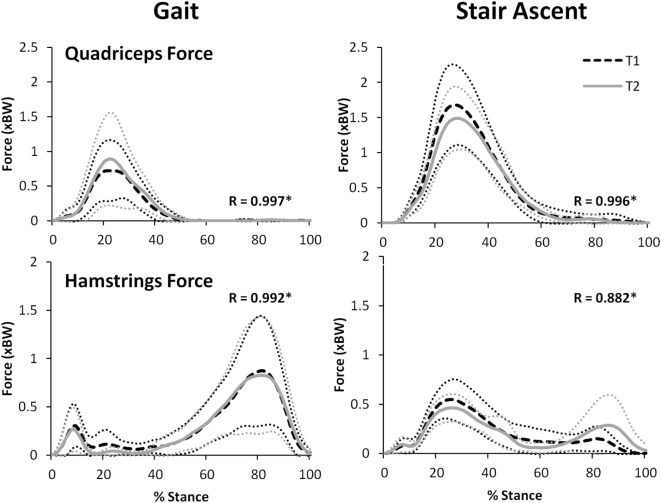
A comparison of group mean quadriceps and hamstring forces from two data collection sessions separated by 24 h. 95% confidence intervals for session one and two are represented by the thin, dotted black and gray lines, respectively. Correlation coefficients (*R*) are also included where * indicates a significant correlation (*p* < 0.05).

Figure 8 illustrates that there was an excellent agreement between the group means of the TFJ and PFJ between T1 and T2. In particular, the correlation coefficients indicate a great deal of similarity between the two waveforms for both walking (*R* = 0.929–0.996) and stair ascent (*R* = 0.950–0.985). Similarly, there was a very high level of agreement in the quadriceps and hamstrings forces predicted during walking and stair ascent (Figure 9) although the level of agreement was not quite as high for the predicted hamstrings forces during stair ascent (quadriceps: *R* = 0.997 walking and *R* = 0.996 stair ascent; hamstrings: *R* = 0.992 walking and *R* = 0.882 stair ascent).

For walking, peak total, lateral, and medial TFJ, and hamstring muscle force, all occurred during late stance (Figures 8 and 9). In contrast, the PFJ and quadriceps muscle force displayed peak force during early stance. For stair ascent, peak total TFJ was achieved during early stance for six participants during T1 and five participants during T2. As for gait, this was predominantly due to a greater medial TFJ. Lateral TFJ reached its peak during late stance. Peak PFJ and quadriceps and hamstrings forces occurred during early stance for all participants.

## Discussion

This study investigated the test–retest reliability of muscle and joint contact forces predicted by FreeBody for gait and stair ascent by quantitative waveform comparisons and by calculating ICC and MDC values based on peak values. The waveform analysis performed evaluated the intra-session reliability of FreeBody, the within-subject inter-session reliability and the inter-session reliability at the cohort level (inter-session reliability of group means).

There was a strong level of agreement between the intra-session values calculated for T1 for all variables for both level walking (mean values: *R* = 0.81–0.93) and stair ascent (mean values: *R* = 0.79–0.97). In addition, consideration of Figures 2 and 3, provides evidence that the waveforms for each trial were qualitatively very similar. Given that each subject would be expected to have some degree of variation in their movement between trials, these data tends to support the contention that the intra-session reliability of FreeBody was good. In practice, it is common to calculate a mean waveform representing the data from several trials in order to minimize the effect of intra-session variability in movement when making inter-session comparisons. The fact that there was a good agreement between the individual trial waveforms intra-session does tend to support this practice, and the remainder of this analysis was based upon the mean waveforms in order to minimize the impact of intra-session variation on the inter-session comparisons.

The within-subject, inter-session comparisons of waveforms also display strong correlations for all variables for walking (mean values: *R* = 0.69–0.91) and stair ascent (mean values: *R* = 0.71–0.97), although the correlations are generally not quite as good as for the intra-session comparisons. Analysis of the subject level correlations and consideration of Figures 4–7 suggests that this is because certain subjects displayed a less strong test–retest performance, rather than this being a trend across all subjects. There is some evidence that at least for one subject this may have been because they were moving differently in T2 (rather than due to measurement error or the model not being reliable). In particular, analysis of the kinematic data revealed that subject RS4 moved differently in the two test sessions. For instance, his peak knee flexion during gait was 18.2° during T1, whereas it was 34.5° during T2 (the mean difference in peak knee flexion between the two trials for the remaining eight subjects was 2.3°).

The ICC values calculated based on the comparison of peak values within subjects varied for the two tasks. For walking, TFJ and hamstring forces had high inter-session reliability; whereas inter-session reliability for PFJ and quadriceps force was poorer. Conversely, for stair ascent, ICC values were higher for PFJ and quadriceps forces in comparison to TFJ and hamstring forces. As a result MDC values were better for TFJ and hamstring force during walking (i.e., the MDCs as a percentage of the mean peak values for total TFJ, medial TFJ, lateral TFJ and hamstring force were 18, 22, 32, and 39%, respectively, whereas the equivalent values for PFJ and quadriceps force were 170 and 168%) and better for PFJ and quadriceps force during stair ascent (i.e., the MDCs for total TFJ, medial TFJ, lateral TFJ, and hamstring force were 49, 59, 110, and 73%, respectively, whereas the values for PFJ and quadriceps force were 28 and 31%).

When comparing the results of this study to the literature, the ICC values for TFJ and hamstring muscle forces during gait are similar to those reported by Gardinier et al. (2013) (0.761–0.922) and Alkjaer et al. (2003) (0.84) suggesting that these predictions are reliable, but the MDC values reported in the current study for total and medial TFJ (0.97–0.99 × BW) are higher than those reported by Gardinier et al. (2013) (0.30–0.66 × BW) and Barrios and Willson (2016) (0.25 × BW). This is likely due to the considerably larger SD reported in this study (1.58–1.76 × BW), and the large confidence intervals seen in Figures 8 and 9 which may be a result of the heterogeneous nature of the subjects studied here.

Minimal detectable changes of 0.43 × BW and 0.99 × BW for lateral and medial TFJ, respectively (as seen in this study), equate to MDCs of approximately 32 and 22% of the mean peak values. These are higher than the MDC that has been reported for gait velocity (Hars et al., 2013; 12%) and the values seen in the Gardinier et al. (2013) study (12 and 18%). However, in contrast they are much better than the MDCs that have been reported for gait parameters like step length and time (Almarwani et al., 2016), knee kinematics (Horsak et al., 2017), peak knee adduction moment (Birmingham et al., 2007), ground reaction force (Fairus et al., 2016), and tibial and femoral accelerations (Turcot et al., 2008). When considered in this context, the performance of FreeBody is similar or better than other current technology.

This study represents the latest incremental step in the process of verification and validation of the FreeBody model. The purpose of this study was to evaluate the test–retest reliability of the model in a population of clinical relevance (i.e., older adults) whose movement might be assumed to be relatively stable (i.e., healthy participants without lower limb pathology). In this way, the reliability of the model itself could be established based upon the assumption that the movement that was being measured was consistent. In addition, this work provides an indication of the MDCs for the model in healthy, older populations. Ultimately however, one goal is to use this model to assess patient populations, whose movement may vary on a day to day basis dependent upon their symptom severity. The MDCs calculated in this study should, therefore, be considered to represent a lower bound for the model when used with this population, and future research should seek to establish MDCs specific to various patient populations.

One example of a potential application for FreeBody is in assessing patients suffering from osteoarthritis of the tibiofemoral joint. It has been demonstrated that there is an association between peak knee adduction moment and knee osteoarthritis (Baliunas et al., 2002; Miyazaki et al., 2002; Foroughi et al., 2009; Maly et al., 2013). This is thought to be because a higher peak knee adduction moment is indicative of greater loading of the medial compartment of the tibiofemoral joint (Foroughi et al., 2009), and thus the peak knee adduction moment is used as a proxy for the relative loading of the tibiofemoral joint. An advantage of musculoskeletal modeling approaches is that a direct estimate of this loading can be calculated (in this study this is presented as the medial TFJ share). In this study, the MDCs for medial TFJ share were lower than for any other variable (4.1 and 5.8% for gait and stair ascent, respectively). When these MDCs are expressed relative to the mean peak values for medial TFJ share, they equate to MDCs of 5–7% (these values are much better than those reported by Gardinier et al. (2013) (22%) and compare favorably with any of the MDCs reported in the literature for variables associated with gait). FreeBody seems to thus be sensitive to a variable that may be clinically important in the evaluation of knee osteoarthritis, and thus may have potential for improving the assessment of knee osteoarthritis risk, severity and disease progression. Of course, future work is required to explore this potential, both in establishing if there is a link between the medial TFJ share and symptoms of osteoarthritis (i.e., establishing the validity of the approach in this population) and to determine the MDC for patients suffering from osteoarthritis (for comparison, Birmingham et al. (2007) found that the MDC for peak knee adduction moments during gait in those who suffer with medial knee OA was 1.0% BW × height or around 40% of the peak value). This work is currently underway in our laboratory.

There was an excellent similarity between the waveforms of the group mean data for all variables investigated during T1 and T2 (*R* = 0.882–0.997). The test–retest reliability of FreeBody at the group mean level is particularly important to establish in order to support the use of the model in research which is based on comparing the group mean waveforms of two or more different groups or cohorts—which is often the approach taken in musculoskeletal modeling research. For instance, FreeBody has been used in two recent research projects that sought to quantify the effects of acute (Parr et al., 2017) and chronic (Czasche et al., 2017) exercise interventions on muscle and joint contact forces. Both of these previous studies demonstrated differences in the group mean waveforms pre and post intervention. The remarkably high correlations between the group mean waveforms of T1 and T2 found in this study tend to support the contention that FreeBody is a reliable tool for comparing group mean data and that differences that might be found pre and post intervention are representative of meaningful changes that can be attributed to the intervention. This study, therefore, supports the use of FreeBody as a tool to investigate the effect of interventions on muscle and joint contact forces at the cohort level.

In conclusion, this study was the first to provide reliability and MDC values for the FreeBody model. This work supports the use of the model to study the effect of interventions at the cohort level in healthy populations. At the subject level, the picture is more complicated. In particular, the within-subject reliability seems to depend on the subject, with some subjects showing good test–retest reliability and others performing less well. If progress is to be made toward a clinical tool that can be used with individual subjects, future research should seek to understand why there is this inter-subject variation in the reliability of the model, and to establish the subject specific detail that is required to rectify this. The MDCs found in this study are generally comparable to or better than traditional measures used to track changes in gait and FreeBody seems to be particularly sensitive to changes in medial TFJ share. This sensitivity should be explored further in the hope of finding a better clinical tool for the assessment of knee osteoarthritis.

## Ethics Statement

All subjects gave written informed consent in accordance with the Declaration of Helsinki. The protocol was approved by the ethics sub-committee of St. Mary’s University.

## Author Contributions

All authors were involved in the conception and design of the study. PP collected all of the data and performed the model and statistical analysis. DC created the musculoskeletal model used in the study. CG verified the statistical approach taken in the study. PP and DC wrote the first draft of the manuscript. All authors were involved in the interpretation of the data, made critical contributions to the manuscript, approved the final version of the manuscript, and take responsibility for the findings of the study.

## Conflict of Interest Statement

The authors declare that the research was conducted in the absence of any commercial or financial relationships that could be construed as a potential conflict of interest.
